# The Influence of Low-Level Laser Therapy on CBCT Radiographic and Biochemical Profiles of Type II Controlled Diabetic Patients After Dental Implant Insertion: A Randomized Case-Control Study

**DOI:** 10.7759/cureus.36559

**Published:** 2023-03-23

**Authors:** Mai S Attia, Gasser M Elewa, Nora Abdelgawad, Radwa M Ismail, Mohamed Hassan Eid, Mohamed M Ghoneim

**Affiliations:** 1 Department of Oral Medicine, Periodontology, Diagnosis, and Radiology, Faculty of Dental Medicine for Girls, Al-Azhar University, Cairo, EGY; 2 Department of Periodontology, Oral Diagnosis, and Oral Radiology, Faculty of Oral and Dental Medicine, Misr International University, Cairo, EGY; 3 Department of Oral Medicine, Diagnosis, and Periodontology, Faculty of Oral and Dental Medicine, Delta University of Science and Technology, Gamasa, EGY; 4 Department of Oral Medicine, Periodontology, and Oral Diagnosis, Faculty of Dentistry, Misr University for Science and Technology (MUST), Cairo, EGY; 5 Department of Oral and Maxillofacial Surgery, Faculty of Dentistry, Suez Canal University, Ismailia, EGY; 6 Department of Oral and Maxillofacial Surgery, Faculty of Dentistry, Sinai University, El-Arish, EGY

**Keywords:** low-level laser therapy, type ii diabetes, osteoprotegerin, implant, bone density, opg

## Abstract

Background

Low-level laser treatment (LLLT) was thought to increase bone quality during osseointegration when combined with dental implants. However, there is no sufficient information on its impact on dental implants in diabetics. Osteoprotegerin (OPG) has been described as a marker for bone turnover to determine implant prognosis. The current research aims to evaluate the effect of low-level laser therapy (LLLT) on bone density (BD) and osteoprotegerin levels in peri-implant crevicular fluid (PICF) in type II diabetic patients.

Methods

This study comprised 40 individuals with type II diabetes mellitus (T2DM). Implants were randomly placed in 20 non-lasered T2DM patients (control) and 20 lasered T2DM patients (LLLT group). At the follow-up stages, BD and OPG levels in the PICF were evaluated in both groups.

Results

Significant variations were shown among control and LLLT groups concerning OPG level and BD (p≤0.001). OPG was significantly decreasing with follow-up points (p≤0.001). There was a significant decrease in OPG with time in both groups with a higher decrease in the control group.

Conclusion

LLLT is promising in controlled T2DM patients due to its outstanding influence on BD and estimated crevicular levels of OPG. Regarding its clinical significance, LLLT significantly improved bone quality during osseointegration on dental implants in T2DM. LLLT is considered potentially important for T2DM patients during implant placement.

Trial registration

The study was registered on ClinicalTrial.gov under registration number NCT05279911 (registration date: March 15, 2022) (https://clinicaltrials.gov/ct2/show/NCT05279911).

## Introduction

Dental implants are widely used and accepted as an efficient management modality for replacing missing teeth in partial or completely edentulous patients [1], with a reported success rate between 90% and 95% [2]. The concept established by Brånemark et al. [3] is primarily responsible for the success of dental implants, stating that it is the interaction between the implant and its surrounding bone as structure and function. In other words, it is a process of bone repair and formation around dental implants due to the osteoblastic and osteoclastic activity of bone [4].

Various studies have reported that good bone density is an essential factor for implant stability to withstand mechanical forces against dental implants. This stability avoids disturbance of both healing around the dental implant and osteointegration to provide a long-term survival rate for dental implants [5,6].

Diabetes mellitus (DM) is a chronic metabolic disorder that has several macro- and microvascular complications because of chronic hyperglycemia. Diabetic patients are prone to developing periodontitis and poor wound healing and are liable for infection [7]. The persistence of hyperglycemia for a long interval of time inhibits bone osteoblastic activity and enhances osteoclastic activities because of inflammatory responses. Also, it causes disturbance in the action of parathyroid hormone, affecting the regulation of Ca and P, which will have an adverse effect on bone healing around dental implants. Accordingly, this may finally affect the long-term implant survival in those patients [8].

Using the level of osteoprotegerin (OPG) as a bone marker to assess implant prognosis has been reported in previous studies, showing a higher concentration of OPG levels in healthy peri-implant tissue than those in cases of peri-implantitis [9,10].

Multiple studies had evaluated the success and failure rates of dental implants in diabetic patients, and the results were conflicting; therefore, further investigations are needed [11-13]. Several studies have demonstrated that low-level laser treatment (LLLT) could enhance the initial stability of osseointegrated implants in animal models [14-16]. LLLT is a sort of phototherapy where infrared (IR) is absorbed by the adjacent tissues, thereby lowering the inflammatory response, bio-stimulating the osteoblastic activity around the application site, and increasing bone production [17]. The photo-biomodulation mechanism is linked with light wavelength penetration, where it has been hypothesized that wavelengths closer to 800 nm may infiltrate deeper, hence enhancing the photobiological process in the surrounding tissues [18].

Nevertheless, there is insufficient evidence about the effects of LLLT on implant steadiness and in explaining the molecular changes in humans, either in healthy situations or in the presence of systemic confounders [19]. Therefore, the current research aims to evaluate the effect of low-level laser therapy (LLLT) on bone density and osteoprotegerin levels in peri-implant crevicular fluid (PICF) in type II diabetic patients.

## Materials and methods

Study design

A randomized case-control study was conducted on a group of 40 patients, out of which 20 were assigned to case and control groups each. A sample size of 40 patients was sufficient to detect an effect size of 0.25 according to power (1-β=0.85) of 85% at a significant probability of p<0.05. The sample size was calculated using the G*Power version 3.1.9.6 software [20].

Ethical approval and consent

The current research was conducted in full conformity with the World Medical Association Declaration of Helsinki. All experimental procedures were performed in accordance with relevant guidelines and regulations. Approval to conduct this study was given by the Institutional Review Board of Misr University for Science and Technology (MUST-IRB) with approval number REC-PD-22-04 on February 9, 2021.

Trial registration

The study was registered on ClinicalTrial.gov under registration number NCT05279911 (registration date: March 15, 2022) (https://clinicaltrials.gov/ct2/show/NCT05279911).

Subjects

The study comprised a total of 40 adults of both sexes who were at least 21 years old. All patients had T2DM under control for more than two years (diagnosed according to the American Diabetes Association criteria) [21]. The participants were willing and able to join in the research. Subjects were selected from the oral diagnosis outpatient clinic of the Faculty of Dental Medicine for Girls, Al-Azhar University, Cairo, Egypt.

Randomization and allocation concealment

**Figure 1:**
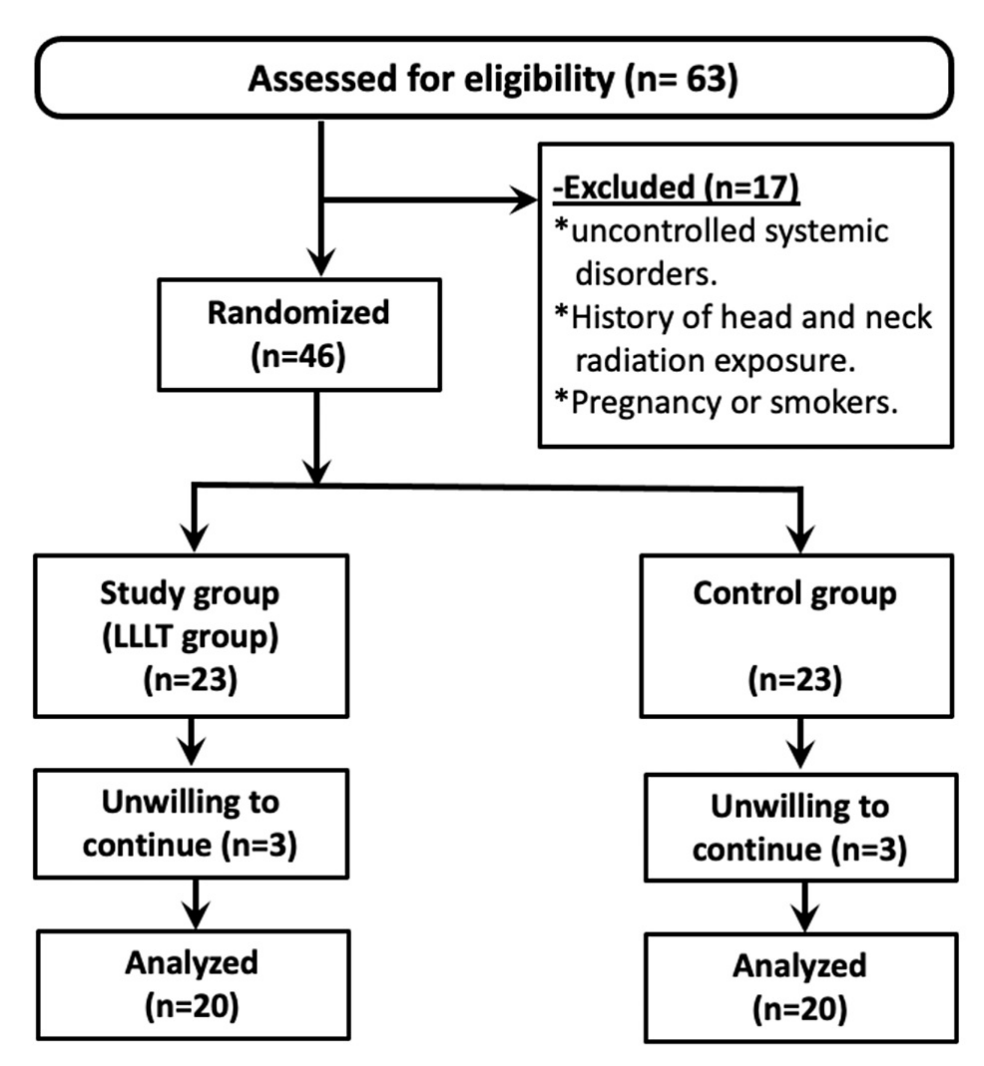
Flowchart of the study population selection. LLLT: low-level laser treatment

Patients were enrolled in the current trial after meeting all eligibility requirements and completing an informed consent form for participation and authorization to use collected data in the current research. They were randomly assigned to one of two groups (control group (conventional implant placement) and LLLT group) based on the method of implant placement (Figure 1). To disguise the randomization process, sequentially numbered envelopes were used. The surgical team was unaware of the sort of intervention until a blinded investigator opened the envelope during the procedure.

Patients who maintained generally consistent glycemic management, with glycosylated hemoglobin A1c (HbA1c) of less than or equal to 6.5% and a difference of less than 1% in at least two HbA1c tests during the preceding six months, which was evaluated by glycosylated hemoglobin A1c (HbA1c) of blood samples, and with a detailed health history acquired using the modified Cornell Medical Index-health questionnaire were included in the current study [22]. Moreover, participants with a partially edentulous posterior mandibular area, bone density between D2 and D3, and neighboring teeth with healthy periodontium and aged between 40 and 48 years were chosen.

However, patients with serious diseases or severe problems from diabetes and any uncontrolled systemic disorders that prohibit implant placement surgery (e.g., uncontrolled diabetes, immunologic disorders, and cardiovascular disorders) were excluded from the current study, as well as those with the need for guided bone regeneration or sinus lift for implant placement and a history of head and neck radiotherapy exposure during the last 12 months. Also, pregnant women, smokers, patients with parafunctional habits and psychiatric issues, and individuals who were unwilling to comply were also eliminated.

Laser setup

Low-level laser (Therapy XT, DMC Group, Sao Carlos, Brazil) (gallium-aluminum-arsenide (Ga-Al-As); l: 808 nm, power density: 50 mW, circular spot diameter: 0.71 cm, spot area: 0.4 cm^2^) in continuous mode was introduced at six-points peri-implant soft tissue contact locations (1.23 minute per application point; dosage per point: 11.0 J) pre- and post-suturing. Two laser spots in the labial area in the proposed implant site (apical and cervical), two laser points in the lingual region (apical and cervical), and two application points in the occlusal direction were determined. Following implant placement and suturing, the LLLT was repeatedly applied to the same sites using the same protocol, resulting in a total dosage of 66.0 J per implant (pre- and post-implanting) [14].

Preoperative phase and instructions

Patients’ personal data were collected, and bone density and the height and width of the edentulous alveolar area were recorded using cone beam computed tomography (CBCT). All patients were advised to maintain oral hygiene using chlorhexidine mouthwash 0.12% for 30-60 seconds, three times a day, for 2-3 weeks.

Surgical protocol

Two-stage implant protocol with the use of a healing cap was used in this study. As the final abutment was not inserted, except after the period of healing, the healing abutment was placed to facilitate the collection of peri-implant crevicular fluid.

Operative phase

Local anesthesia was performed at the surgical site; then, the surgical site was cleansed using betadine (povidone-iodine). The incision was made using blade number 15c on the midline of the crestal mucosal gingiva of the edentulous area (implant site) and extended two adjacent teeth by sulcular incision, and then, a full-thickness mucoperiosteal flap was reflected to expose the ridge.

The dental implants (Multysystem, Lissone, Italy) used in this study were of root-form threaded and internal hex design. The implants were made of pure titanium with lengths ranging from 11 to 13 mm and a diameter ranging from 3.5 to 5 mm.

**Figure 2:**
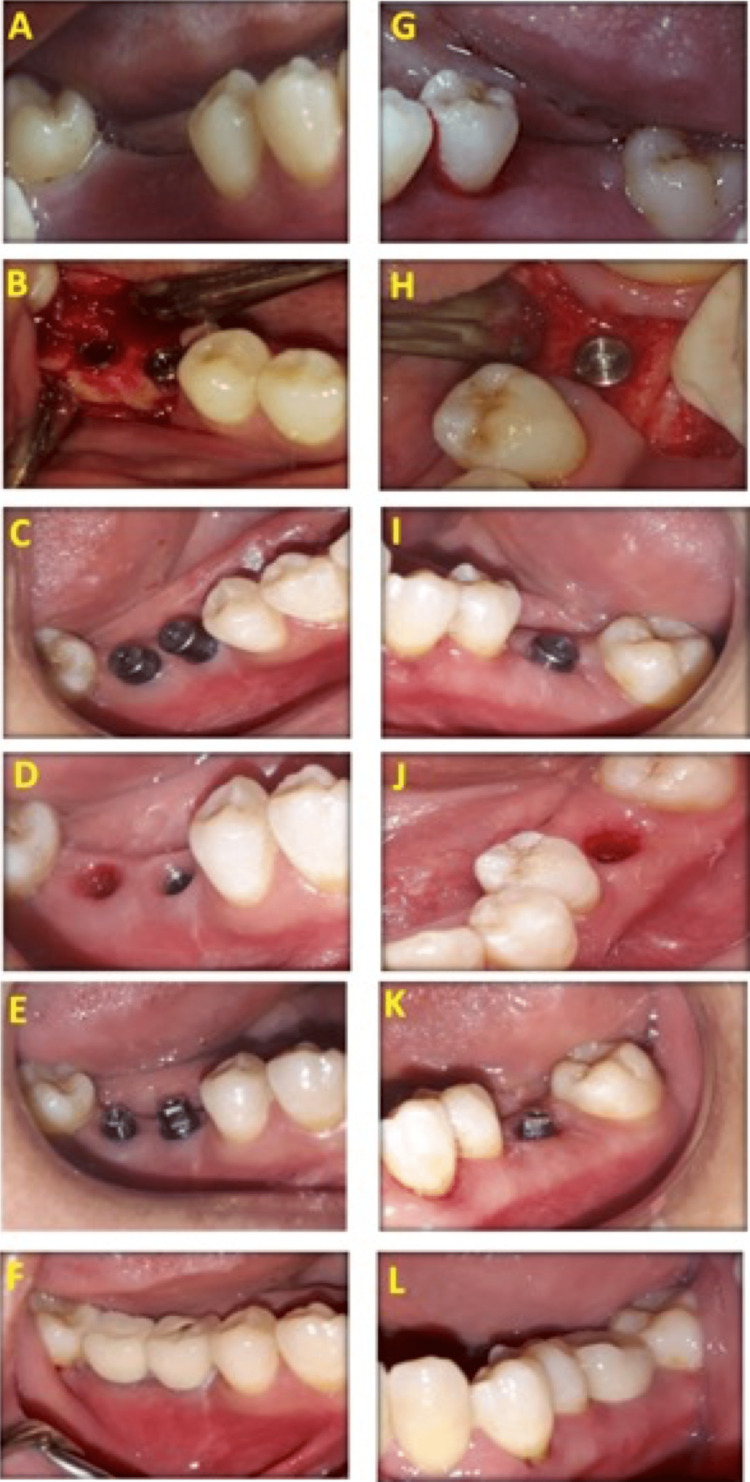
Photographs of the control group (A-F) and LLLT group (G-L). Preoperative (A,G), fixture placement at the crestal level (B,H), after three months showing healing abutment (C,I), tissue profile after the removal of the healing abutment (D,J), final abutment construction (E,K), and after crown cementation (F,L) in the control and LLLT groups, respectively LLLT: low-level laser treatment

Osteotomy was presented in detail in Figures 2A-2F for the control group and Figures 2G-2L for the LLLT group. Osteotomy sites were determined (Figure 2A and Figure 2G), and osteotomy sites were first performed using a pilot drill, followed by drills sequentially until the required diameter of the fixture was reached (Figure 2B and Figure 2H). Drilling occurs under copious internal irrigation with saline deep inside the bone to avoid overheating of bone over 47°C. The osteotomy site was prepared with a 0.5 mm diameter less than the fixture diameter as recommended by implant system instruction, then implants were introduced in its site by fixture driver, and finally, a torque gauge ratchet was used to place the implant in its final position (Figures 2B-2C and Figure 2H-2I). The fixture was placed at a crestal position, followed by the screwing of a polished healing abutment, 2-4 mm in height, to the implant body (Figure 2B and Figure 2H). The tissue profile after the removal of the healing abutment in the control group was presented in Figure 2D and in the LLLT group in Figure 2J. The final abutment construction is shown in Figure 2E for the control group and Figure 2K for the LLLT group.

Soft tissues were sutured finally with interrupted silk 4/0 sutures (silk suturing was done with interrupted sutures using non-resorbable sutures, 4/0 black silk) and removed one week postoperatively. Chlorhexidine mouthwashes (0.12%) were used three times a day before surgery to reduce the bacterial load and for two weeks after surgery twice per day [23]. Ibuprofen 600 mg tablets were prescribed as an analgesic, if needed.

Assessment parameters

Cone Beam Analysis

CBCT was taken for each patient using the Planmeca machine (ProMax 3D Mid, Planmeca, Helsinki, Finland). The tube voltage was 90 kV, and the tube current was 12 Ma according to the field of view of pulse exposure. For the implant treatment plan, CBCT data was a valuable resource for information that enhances the treatment plan as a linear measurement including bone density. The CBCT was taken at the baseline and six months following implant placement to evaluate bone density.

**Figure 3:**
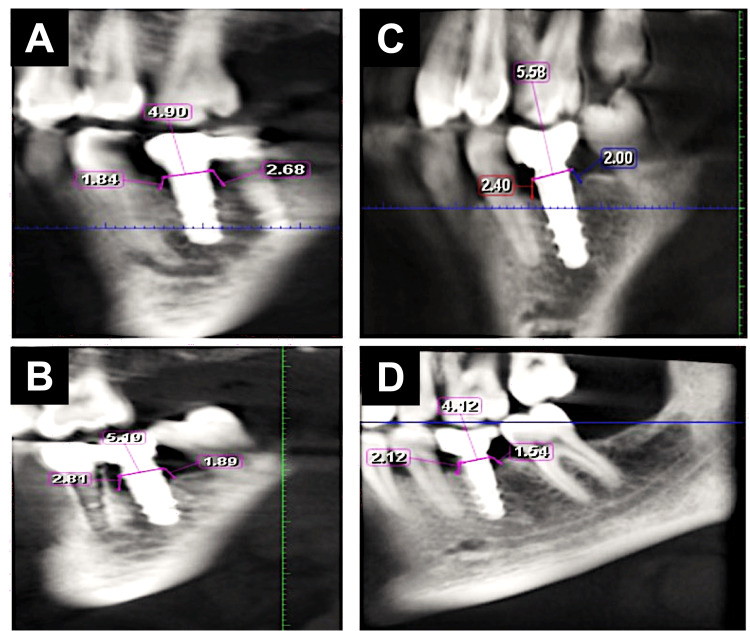
Radiographic evaluation of bone density using CBCT six months after fixture insertion in the control group (A,B) and LLLT group (C,D). The cross-sectional view along the middle of the implant was used to measure the gray values in three regions buccally and lingually. The total length of the implant was measured and then divided into four parts in total; the first part is the most coronal 2 mm of the bone representing compact bone, and the rest of the distance was divided into three parts representing the cervical, middle, and apical thirds. CBCT: cone beam computed tomography, LLLT: low-level laser treatment

Bone density: The bone density value (as gray value) was determined using the BlueSkyPlan software (BlueSkyPlan, Libertyville, IL, USA) that automatically illustrated the difference in the gray value in numbers by moving the pointer from one region to another. Bone density was taken at a fixed point on the software at baseline and after six months of implant placement. The bone density was taken into two planes: coronal and sagittal. For each one, two lines were drawn parallel to the entire length of the implant, and each line was divided into three areas (coronal, middle, and apical thirds). These three lines were recorded at a fixed point away parallelly from the implant to be away from the titanium artifact at the bone-implant interface. The bone density value around the implant was measured automatically by the CBCT software, which was recorded in Hounsfield units. Radiographic evaluation of bone density (BD) was performed using CBCT twice, before fixture insertion and six months after fixture insertion (Figures 3A-3D).

Biochemical Evaluation

Peri-implant crevicular fluid (PICF) samples were collected from participants at intervals of one week, one month, three months, and four months to evaluate OPG levels. Samples were collected using a paper point after complete isolation of the implant site, and soft tissue was gently dried using an air syringe. Paper points were gently introduced into the newly formed gingival sulcus until mild resistance was sensed and left in place for 30 seconds. Paper points that are contaminated with saliva or blood were excluded. Paper points were placed then in Eppendorf vials, which were kept at a temperature of -80°C until processing. OPG was determined in PICF using a human osteoprotegerin instant ELISA kit (BMS2021INST) provided by Bioscience (Bender MedSystems GmbH, Vienna, Austria).

Statistical analysis

Statistical tests were conducted using the Statistical Package for the Social Sciences (SPSS) software version 28.0 for macOS (IBM SPSS Statistics, Armonk, NY, USA) at a significance of 0.05. Kolmogorov-Smirnov test was used to assess the normality of study variables, whether parametric or nonparametric, and data were normally distributed, i.e., parametric data. Data were presented as mean and standard deviation (SD) for parametric data. Student’s t-test was used for comparison between the two groups. The differences between the two groups at two time points were evaluated using repeated measures ANOVA at 0.05 level. The difference between baseline and six months postoperative is evaluated using paired samples t-test [24]. The difference between time points (one week, one month, three months, and four months) was evaluated using repeated measures ANOVA.

## Results

Patients’ criteria

The present study included 40 controlled T2DM patients with missing posterior teeth; the control group included 11 females and nine males, while the LLLT group included eight females and 12 males. All patients were allocated to their group and established one-stage dental implant replacement for missing posterior teeth. The age ranged between 40 and 44 years old, with an average(±SD) of 42.8±1.6 and 43.1±1.4 in the control and LLLT groups, respectively. The difference in age between the two groups is nonsignificant as revealed by the independent samples t-test.

During the study, patients in the two groups showed consistent and comparable oral hygiene standards. All patients completed the proposed clinical follow-up visits. Regarding treatment tolerance, the two treatment modalities were well tolerated by participating patients without any complications or side effects. Throughout the research period, none of the implants revealed any clinical symptoms of peri-implant infection or detectable movement.

Bone density (BD)

**Figure 4:**
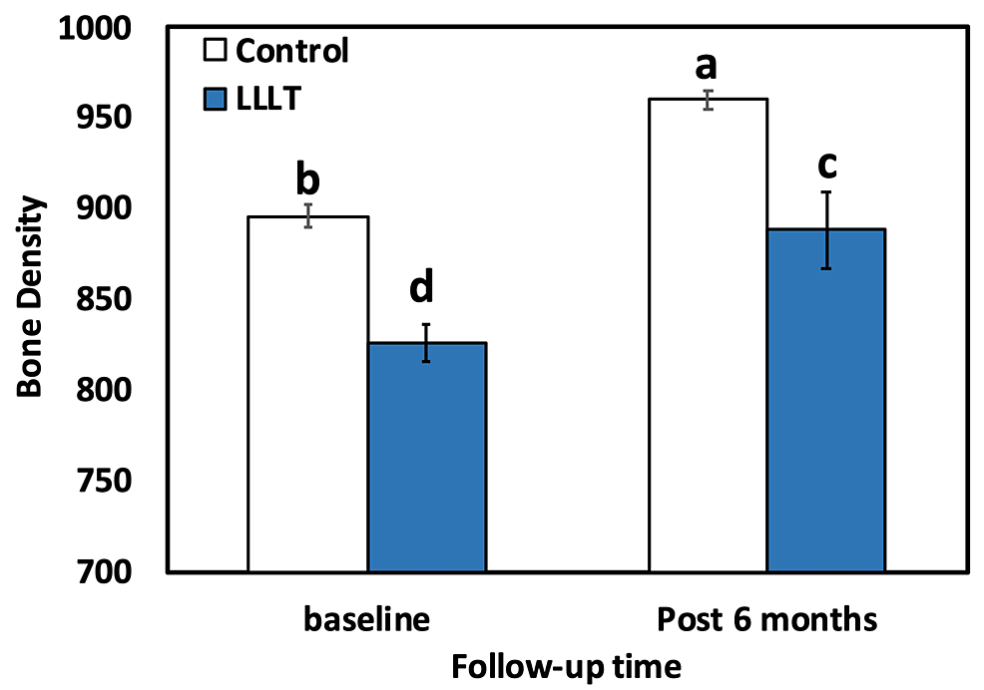
Bar chart comparing the mean BD level for both groups (control and LLLT). Bars with different letters are significantly different according to DMRTs. LLLT: low-level laser treatment, BD: bone density, DMRTs: Duncan’s multiple range test

For both groups (control and LLLT), the intragroup comparison (paired t-test) result showed statistically significant (p<0.001) differences between two baselines, 896.00±28.68 in the LLLT group and 826.00±44.47 in the control group. After six months, the result was in favor of the LLLT group, which has a higher mean BD of 960.00±22.71, compared to the control group with 888.25±92.64. The results of intergroup comparison using the independent-t-test showed a highly significant (p<0.001) distinction between the control and LLLT groups at different time intervals (baseline and six months after) (Table 1, Figure 4). 

**Table 1:** Comparative evaluation of mean BD between the two groups. Means followed by different letters are significantly different according to DMRT at 0.05 level. BD: bone density, LLLT: low-level laser treatment, DMRTs: Duncan’s multiple range test

Follow-up time point	Bone density/group		p-value
	LLLT	Control	
Baseline	896.0±28.68^b^	826.00±44.47^d^	<0.001
Post six months	960.0±22.71^a^	888.25±92.64^c^	<0.001
p-value	<0.001	<0.001	

Osteoprotegerin (OPG) levels

**Figure 5:**
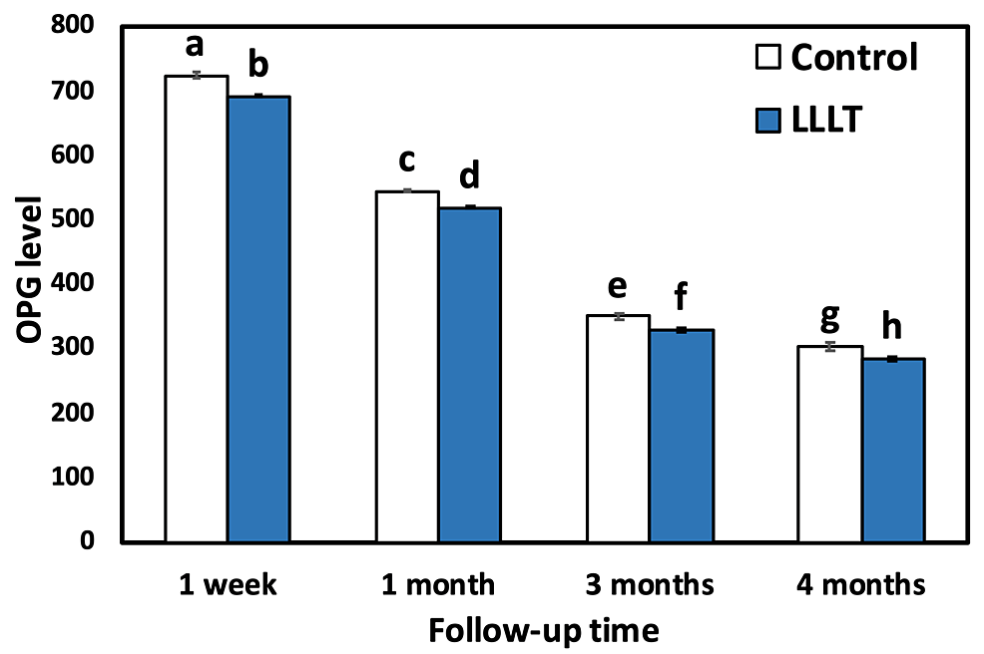
Bar chart comparing the mean OPG level for both groups (control and LLLT) at different time intervals. Bars with different letters are significantly different according to DMRT. OPG: osteoprotegerin, LLLT: low-level laser treatment, DMRTs: Duncan’s multiple range test

**Figure 6:**
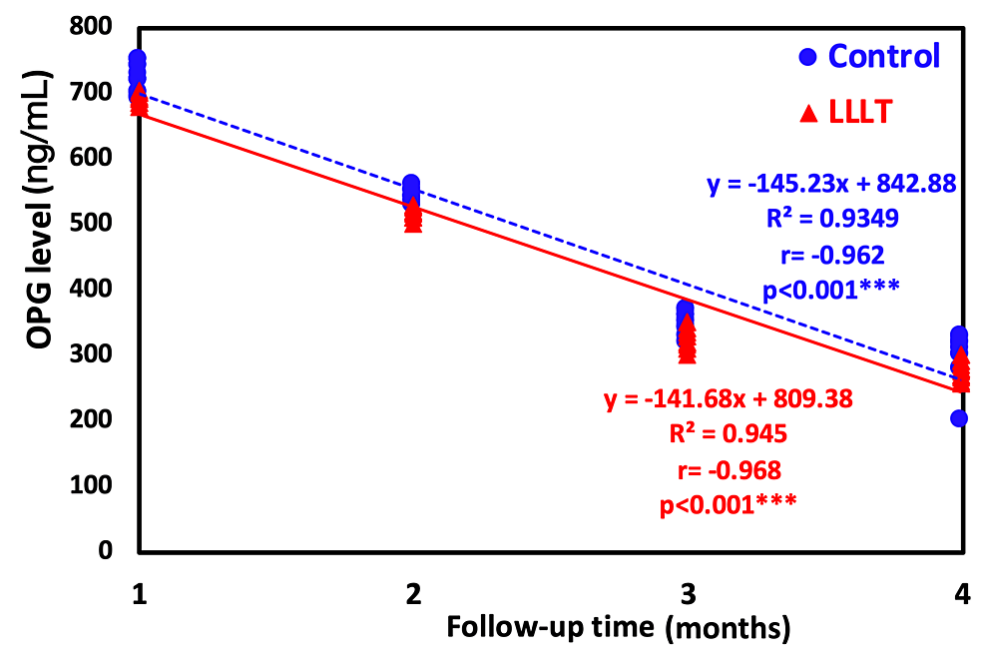
Regression trendline showing the relationship between time of investigation and OPG level. OPG: osteoprotegerin, LLLT: low-level laser treatment

The outcomes of intragroup comparison using repeated measures ANOVA for the control group showed that there was a statistically significant (p<0.001) difference (decrease) in the mean OPG level between all follow-up time points. The use of Duncan’s multiple range test (DMRT) revealed that there was a significant difference between one week and all follow-ups as well as between one month, three months, and four months (p<0.001). Also, there was a significant difference between three and four months. For the LLLT group, there was a statistically significant difference (decrease) in the mean OPG level between all follow-up time points as revealed by repeated measures ANOVA and between one week and all follow-ups as well as between one month, three months, and four months (p<0.001). Also, there was a significant difference between three and four months (Table 2, Figure 5 and Figure 6).

**Table 2:** Comparative evaluation of the mean OPG level between the two groups. Means followed by different letters are significantly different according to DMRT at 0.05 level. OPG: osteoprotegerin, SD: standard deviation, LLLT: low-level laser treatment, ANOVA: analysis of variance, DMRTs: Duncan’s multiple range test

Follow-up time point	OPG level (mean±SD)/group		p-value
	LLLT	Control	
1 week	722.75±20.36^a^	691.25±7.05^b^	<0.001
1 month	543.75±10.50^c^	518.75±8.56^d^	<0.001
3 months	349.25±15.83^e^	328.25±14.35^f^	<0.001
4 months	303.50±29.29^g^	282.50±13.91^h^	<0.001
ANOVA	<0.001	<0.001	

The results of the intergroup comparison using an independent t-test indicated a statistically highly significant (p<0.001) difference between the control group and the LLLT group at different time intervals. The results of the two-way repeated measures ANOVA revealed overall highly significant (p<0.001) differences between the groups (control and study) and between time points. These results are comparable with the results of the independent t-test mentioned above (Table 2).

Figure 6 represents a regression analysis with a trendline showing the relationship between the time of investigation and the OPG level. The regression trendline revealed a highly significant decrease in the OPG level with time either in the LLLT group (r=-0.92; p<0.0001) or the control group (r=-0.968; p<0.0001). However, the control group showed the highest decrease rate by having a higher negative correlation coefficient.

## Discussion

The results from the current study indicated that LLLT improved bone density levels, promoted bone repair, and enhanced bone structure significantly with a follow-up period of six months with a significantly lower OPG level at LLLT in type II diabetic patients. Both control and LLLT groups showed a significant decrease in OPG levels; however, the LLLT group showed lower values.

Due to lower protein metabolism, the repair process of hard and soft tissues is slowed in diabetes patients compared to healthy people. It is also influenced by decreased neutrophil function. Due to these factors, DM has occasionally been considered a contraindication for dental implant placement [25]. Bone remodeling is an important characteristic of implant existence to various functions employed on the implant restoration and its associated bone, particularly in diabetics, because the glycemic control in relation to the development of microvascular and macrovascular problems is well recognized [26] and may compromise healing capacity [27]. In this regard, LLLT was proven to increase the density of bone lamella meshwork of compact bone and also increase its strength when directed on the tibia of streptozotocin-induced diabetic rats [28].

Therefore, it may be assumed that LLLT might increase bone integration, therefore boosting implant stability and lowering the aforementioned negative side effects. Given that OPG levels have been identified as a biomarker for bone turnover used to predict implant prognosis [9], the current research was directed to evaluate bone density and OPG levels in the PICF in lasered versus non-lasered controlled T2DM patients. This is also one of the few randomized controlled experiments that looked at the effects of LLLT on dental implants in diabetic individuals [29]. The increased BD at six months in both groups can be explained in light of the findings that confirmed that BD increased around dental implants after placement due to an increase in the mineralization of tissue [30].

Endosseous healing relies on osteogenic cell migration to the peri-implant region during the healing phase [29,31]. So, the LLLT application could be proposed to have a role in early bone formation throughout the osseointegration time as it facilitates bone healing by means of increased angiogenesis and collagen fiber deposition [29,32]. LLLT additionally helps in bone cell proliferation and differentiation via enhanced BMPs and transcription factor expression that is linked to osteoblast differentiation [33]. Our intergroup findings comparing BD in both the LLLT and control groups were in line with this aforementioned discussion. The LLLT group showed higher statistically significant BD values around implants compared to the control group (p≤0.05) at six months.

Regarding OPG, in the first week, both groups reflected similar levels. The highest OPG level recorded at this time point confirmed the fact that high bone remodeling occurs after implant insertion, and the peak of this remodeling was reported to be at seven days of fixture insertion [34]. The OPG concentration level increased at this stage as it is crucial in the control of the bone remodeling process by inhibiting osteoclast maturation and activation to prevent bone resorption [26]. This was followed by a subsequent significant decrease in the OPG level in both groups at all follow-up time intervals (one month, three months, and four months). These results are attributed to the changes in bone remodeling that occurred around dental implants [26].

On the other hand, a significantly decreased OPG level was detected in the LLLT group as compared to the control group. This result can be well explained based on the biological effects of LLLT on inflammation control and modulation of bone resorption, steps that upregulate the bone remodeling process [35].

Furthermore, the majority of experimental research indicates that bone growth surrounding dental implants would be inadequate and delayed. Consequently, the newly produced bone was immature and poorly structured, preventing implant contact [7]. Besides, the bone tissue was not as well structured around dental implants in diabetic rats [36]. This explanation is confirmed by the intergroup results of the LLLT group and the regression analysis.

The receptor activator of nuclear factor-kappa B (RANK), RANK ligand (RANKL), and OPG have a crucial role in triggering bone remodeling [37]. As essential osteoclastogenesis regulating molecules, RANKL and its two receptors, RANK and OPG, have been demonstrated to be involved in the remodeling process. In bone, osteoblast cell lineage and periodontal ligament (PDL) cells express RANKL [38], and as an osteoclast differentiation factor, it acts directly after attaching to the RANK receptor on the cell surface of osteoclast lineage cells. This interaction accelerates the maturation of hematopoietic osteoclast precursors. Osteoblasts and other cells create OPG, which functions as a decoy receptor that interferes with RANKL for attaching to its receptor RANK. This affects bone resorption by inhibiting osteoclast growth and differentiation. Any inconsistency in the interaction between OPG, RANK, and RANKL proteins might result in abnormalities in the mineralized tissue created [37].

Recently, the effects of LLLT on alveolar socket osteoclastogenesis signaling were assessed in rats after tooth extraction [39]. RANK and RANKL gene expression was elevated during the earliest phases of healing in the laser-treated group, but OPG gene expression was increased throughout the duration of the research. LLLT seems to promote osteoclastogenesis-favorable gene expression during the first stages of healing, while formation bone stimulation is delivered throughout the duration of the research. This novel theory is consistent with our LLLT group results. Ultimately, through the satisfactory results shown in this current study, to determine the long-term survival of implants in various groups of diabetic patients, further research and clinical studies must be conducted over a longer period.

In the last decades, the use of LLLT has been widely studied in dentistry [40-43]. In oral implantology, some studies have assessed the use of LLLT to improve the primary stability at the early stages of osseointegration [16]; however, most of these studies were performed in animal models. As stated earlier, the last systematic review on this topic suggested that there is a lack of evidence about the influence of LLLT on implant stability in humans [19]. In this way, a recent clinical trial on orthodontic mini-implants has suggested that the secondary stability was increased when LLLT was applied [44]. Our findings suggest that LLLT is promising in controlled T2DM patients due to its outstanding influence on BD and estimated crevicular levels of OPG.

The main limitation of studying LLLT application is only on the day of the surgery since constant LLLT could have more effect on the studied outcomes. Therefore, the use of LLLT to promote implant osseointegration needs further in vitro and in vivo studies that should focus on the mechanisms involved in the desirable and undesirable effects of irradiation.

## Conclusions

In conclusion, the results of the current study indicated that LLLT improved bone density levels, promoted bone repair, and enhanced bone structure significantly with a follow-up period of six months with a significantly lower OPG level in type II diabetic patients. Both control and LLLT groups showed a significant decrease in OPG levels; however, the LLLT group showed lower values. The application of LLLT seems to have an effective adjunctive therapy in improving bone density and osteoprotegerin (OPG) around placed dental implants in type II diabetics. The significant change in OPG levels would be considered a valuable indicator of the osteogenic efficacy of using LLLT around dental implants. Moreover, OPG can be used as a good bone formation biomarker. Finally, LLLT is a promising option to be used in controlled T2DM patients due to its outstanding influence on bone density and estimated crevicular levels of OPG. Therefore, the use of LLLT to promote implant osseointegration needs further in vitro and in vivo studies, which should investigate the mechanisms involved in the desirable and undesirable effects of laser irradiation.
